# The association between medication use and lifestyle factors in independently living older people: impact of Mediterranean diet and physical activity

**DOI:** 10.1016/j.jarlif.2025.100041

**Published:** 2025-11-07

**Authors:** Lieke Roeke, Greg Kennedy, Denny Meyer, Michael Kingsley, Catherine Itsiopoulos, Leonie Segal, Anne-Marie Minihane, Karen J Murphy, Tuan Anh Nguyen, Jeffery M Reddan, Joris C Verster, Andrew Pipingas

**Affiliations:** a Centre for Mental Health and Brain Sciences, Swinburne University, Melbourne, VIC 3122, Australia; b Division of Pharmacology, Utrecht Institute for Pharmaceutical Sciences, Utrecht University, 3584CG Utrecht, the Netherlands; c Department of Clinical Pharmacy and Toxicology, Maastricht University Medical Centre+, Maastricht, the Netherlands; d Department of Exercise Sciences, University of Auckland, New Zealand; e Holsworth Research Initiative, La Trobe University, Australia; f Nutrition and Preventive Medicine, Norwich Medical School University of East Anglia, Norwich, UK; g Alliance for Research in Exercise, Nutrition and Activity (ARENA), Clinical Health Sciences, University of South Australia, Australia; h School of Health and Biomedicine STEM College, RMIT University, Australia; i Health Economics and Social Policy, Allied Health and Human Movement, University of South Australia, Australia; j Social Gerontology Division, National Ageing Research Institute, Australia; k Department of Psychological Sciences, School of Health Sciences, Swinburne University of Technology, Australia; l Quality Use of Medicines and Pharmacy Research Centre, UniSA Clinical & Health Sciences, University of South Australia, Australia; m Cognitive Neurophysiology, Department of Child and Adolescent Psychiatry, Faculty of Medicine, TU Dresden, D-01307 Dresden, Germany

**Keywords:** Older adults, Medication use, Lifestyle factors, Mediterranean diet, Physical activity

## Abstract

**Objectives:**

Adherence to the Mediterranean diet (MedDiet) and engagement in physical activity (PA) are associated with a lower risk of chronic disease, yet their relationship with medication use in older adults is less clear. This study examined cross-sectional associations between MedDiet adherence, PA levels, and medication use, including polypharmacy, in independently living older Australians.

**Design:**

A cross-sectional observational study

**Setting and participants:**

Baseline data were drawn from the MedWalk trial, including 161 participants (119 females, 42 males; aged 60–90 years) residing in independent living facilities in Adelaide and Melbourne, and the wider Melbourne community.

**Measurements:**

Adherence to the MedDiet was assessed using the 14-item Mediterranean Diet Adherence Screener (MEDAS). PA was objectively measured via accelerometry and categorized as low (<150 min/week), moderate (150–<300 min/week), or high (≥300 min/week) of moderate-to-vigorous PA. Medication use was self-reported and coded using Anatomical Therapeutic Chemical (ATC) classifications. Polypharmacy was defined as use of ≥5 medications. Logistic and negative binomial regression analyses were adjusted for age, sex, education, and BMI.

**Results:**

Moderate and high PA were associated with significantly lower odds of polypharmacy compared with low PA (AOR=0.37, *p* = 0.047; AOR=0.08, *p* = 0.022, respectively). High PA was also associated with a reduced total number of medications (exp(B)=0.44, *p* = 0.018) and fewer cardiovascular medications (AOR=0.29, *p* = 0.042). Each additional point in MedDiet adherence was associated with reduced use of alimentary tract and metabolism medications (AOR=0.78, *p* = 0.016), though no association was found with total medication use or polypharmacy.

**Conclusion:**

In this cross-sectional study, higher PA was consistently associated with reduced polypharmacy and fewer medications, while greater MedDiet adherence was linked to reduced use of metabolic medications. Lifestyle practices may influence medication use patterns in later life. Larger longitudinal studies are needed to clarify causality and inform strategies to reduce polypharmacy through promotion of healthy lifestyle behaviors.

## Highlights

• Moderate and high PA are linked to lower odds of polypharmacy in independently living older people.
• High PA is associated with fewer total medicatios and reduced cardiovascular drug use.
• Higher MedDiet scores are linked to reduced use of alimentary tract and metabolism medications.
• Encouraging a healthy lifestyle through PA or closely following a MedDiet might help reduce the development of chronic diseases and medication use in older adults.

## 1 Introduction

People aged 65 years and older represent the fastest growing demographic worldwide, with projections indicating their numbers will more than double to over two billion by 2050 [1]. With advancing age, the prevalence of chronic conditions increases substantially. In Australia, nearly half the population (47 %) had at least one chronic condition in 2020–2021, and more than half (51 %) of those aged 65 years and over had two or more, compared with only 12 % in adults aged 15–44 years [2]. Chronic diseases often co-occur, leading to complex health profiles that are typically managed through pharmacotherapy. Consequently, polypharmacy, commonly defined as the concurrent use of five or more medications, is highly prevalent in older adults, affecting nearly one million Australians aged over 70 in 2017 [3,4]. While medications are critical for disease management, polypharmacy is associated with adverse drug reactions, medication errors, functional decline, falls, hospitalisation, and increased mortality [3]. Reducing inappropriate polypharmacy has therefore been identified as a global health priority, with the World Health Organization (WHO) calling for a 50 % reduction in iatrogenic medication-related harm as part of its third Global Patient Safety Challenge [5]. Both physical activity (PA) and the Mediterranean diet (MedDiet), independently reduce the chances of developing chronic diseases and promote longevity [[6], [7], [8]]. Regular moderate-to-vigorous physical activity (MVPA) is linked to numerous health benefits, including reduced risks of cardiovascular disease (CVD), cancer, and all-cause mortality [6,7]. Higher volumes of PA, particularly at moderate intensity, are associated with lower rates of incident CVD in both men and women [9]. PA aids in weight reduction, lowers blood pressure, and improves blood lipid profiles by increasing high-density lipoprotein cholesterol and reducing triglycerides [6,7,9,10]. Individuals with chronic conditions can still experience preventive health benefits by engaging in higher levels of PA [7].

The MedDiet is widely regarded as a healthier alternative to the Western diet [11]. Characterized by high consumption of extra virgin olive oil, fruits, nuts, vegetables, cereals, moderate intake of fish and poultry, low intake of dairy foods, red and processed meats, sweets, and moderate red wine with meals, the MedDiet provides high intakes of antioxidant vitamins, fibers, unsaturated fats, and phytochemicals like sterols and flavonoids [11]. Promoted globally for its health benefits, the MedDiet is consistently associated with lower chronic disease levels and increased longevity. An umbrella review of meta-analyses, including over 12.8 million participants, found that greater adherence to the MedDiet reduces the risk of mortality, cardiovascular disease, cancer, neurodegenerative diseases, and type 2 diabetes [8].

Unfortunately, the independent and additive benefits of lifestyle factors, such as maintaining a healthy diet and regular PA, are often underemphasized in treating or preventing chronic diseases. Additionally, the relationship between adherence to a healthy diet like the MedDiet, PA levels, and medication use in older adults remains unclear. The aim of this study was to investigate the association between lifestyle factors and medication use, in particular polypharmacy and number of medications used. We hypothesized that greater adherence to the MedDiet, or higher levels of PA would be associated with reduced medication use and polypharmacy. Furthermore, we hypothesized that higher adherence to these lifestyle factors would be associated with reduced use of specific medication groups like alimentary tract and metabolism, blood and blood forming organs, cardiovascular system, musculoskeletal, and the nervous system.

## 2 Methods

### 2.1 Study design

Cross-sectional associations were analyzed between adherence to the Mediterranean diet, physical activity levels, and medication use using baseline data from the MedWalk clinical trial. This trial investigated the effects of Mediterranean diet and walking intervention on cognitive decline in independently living older Australians [12].

The study was approved by the Swinburne University Human Research Ethics Committee (project number 2022/009) and registered with the Australian New Zealand Clinical Trials Registry (ACTRN12620000978965). The study was conducted in accordance with the Declaration of Helsinki, and its latest amendments, and written informed consent was obtained from all participants. Given the cross-sectional nature of the current study STROBE guidelines for reporting were applied.

### 2.2 Participants

Participants aged 60–90 years, residing in independent living facilities (ILF) (i.e., without the need for external support for their daily living activities) in Adelaide and ILFs and the general community in Melbourne, Australia, were recruited for the MedWalk trial. A total of 161 participants (119 females, 42 males) were included in this study's baseline analysis. A flow diagram is shown in Table S1.

Participants were fluent in written and spoken English, able to walk independently, and free from major physical ailments that prevent regular walking. Potential participants were excluded from MedWalk if they had suspected cognitive impairment (defined as a score <24 on the Mini Mental State Examination) or depression (defined as a score >10 on the 15 items Geriatric Depression Scale), had been diagnosed with dementia or other forms of cognitive impairment, had a history of stroke or other neurological condition that causes significant functional or cognitive issues, or had been diagnosed with a mental health condition that is uncontrolled (by medication or intervention) which has a significant impact on daily life, use cholinesterase inhibitors or had a diagnosed allergy or intolerance of food groups that would prevent adherence to a Mediterranean diet. Finally, those who reported participating (on average) in >150 min of moderate-to-vigorous leisure time physical activity per week and had a high MedDiet adherence score (defined as a score ≥10 on the 14-item PREDIMED diet questionnaire) were also excluded.

### 2.3 Medication assessment

Participants recorded all prescription and over-the-counter medications taken in the two weeks before screening by completing a questionnaire at home. They provided details including the medication name, reason for use, dosage, frequency, and start and stop dates. Medications were coded based WHO Anatomical Therapeutic Chemical (ATC) classification and participants’ self-reported reasons for medication use (Table S2) [13]. The ATC classification was based on what the medication was prescribed for. Only medications related to the alimentary tract and metabolism, blood and blood-forming organs, cardiovascular, musculoskeletal, and nervous systems were assessed, given their relevance to the MedDiet and PA based on plausible physiological mechanisms and associations of these medications [[6], [7], [8], [9], [10],[14], [15], [16]]. The primary outcome polypharmacy was defined as more than five medications [17]. Secondary outcomes included the total number of medications used and use of individual medication groups (alimentary tract and metabolism, blood and blood-forming organs, cardiovascular system, musculoskeletal system, and nervous system medications).

### 2.4 Dietary assessment

The Mediterranean Diet Adherence Screener (MEDAS) is a validated 14-item tool used to assess adherence to the MedDiet [16]. It includes two questions on food habits and twelve on food frequency. Each of the 14 items are scored 1 or 0, depending on whether participants adhere to each MedDiet component or not. The MEDAS items and the criteria for scoring 1 point are shown in Table S3. If these conditions were not met, an item was assigned a score of 0. The resulting MEDAS-derived MedDiet score ranged from 0 to 14 , with a total score of ≥10 indicating high adherence [16]. Our Modified MEDAS recommends ≥3 tablespoons/day of extra virgin olive oil (EVOO), unlike the PREDIMED trial’s ≥4 tablespoons/day, as EVOO is less commonly consumed in Australia [18]. This adjustment aims to improve adherence and sustainability in the MedWalk intervention. The MedLey trial used a lower recommendation of ≥1 tablespoon/day and reported cardiovascular health benefits from MedDiet adherence [18].

### 2.5 Physical activity assessment

PA was assessed using a tri-axial accelerometer (wGT3X-BT; Actigraph, Pensacola, FL, USA) worn on the right hip for eight days, excluding sleep, swimming, and showering. Devices recorded raw accelerations at 100 Hz, and data were analyzed using the manufacturer’s software (Actilife v7; Actigraph, Pensacola, FL, USA). Non-wear time was identified and removed automatically after visual verification against non-wear times that were recorded by participants in a diary [19]. Participants were included if they wore the device for at least 10 h/day on 4 days, including one weekend day [19]. Based on previous literature, moderate-to-vigorous physical activity (MVPA) was defined as ≥2751 Counts Per Minute (CPM), selected as an age-specific cutpoint where VM CPM is equivalent to 3 METS (moderate intensity exercise), with weekly totals used to categorize participants as ‘low’ (<150 min/week), ‘moderate’ (150 to <300 min/week), or ‘high’ (≥300 min/week) [20].

### 2.6 Covariates

Age, sex, education, and body mass index (BMI, kg/m^2^) were recorded as part of the study requirements. Age (in years) was calculated from the self-reported birth date. Age, sex, BMI, and education level were included as potential confounders. Education may serve as a proxy for socioeconomic status, and BMI is directly related to medication use and health outcomes.

### 2.7 Statistical analysis

Descriptive statistics were used to summarize participant characteristics and study outcomes. Table 1 presents baseline characteristics stratified by polypharmacy status (≥5 medications vs <5 medications), Univariate analyses were performed to examine associations between each independent variable and polypharmacy status. Age, sex, education level and BMI were included in the multivariate models described below.

**Table 1:** Baseline characteristics.

	Total (*n* = 161)	Polypharmacy* Yes (*n* = 50)	Polypharmacy* No (*n* = 110)	*p*-value
**Demographic & Morphometrics**				
Age (mean ± SD)	74.93 (5.94)	76.88 (5.98)	74.09 (5.74)	0.006
Sex ( % female)	73.9 %	74.0 %	73.6 %	0.961
Education level				0.696
Primary school	17	7	10	
Secondary school	42	13	29	
Diploma	46	16	30	
Undergraduate degree	33	10	23	
Postgraduate degree	20	4	16	
Missing	3	0	2	
				
BMI, kg/m^2^	29.21 (5.41)	31.05 (5.85)	28.39 (5.03)	0.004
**Diet**				
MedDiet-score, max 14 points (mean ± SD)	5.67 (1.93)	5.58 (1.81)	5.71 (2.0)	0.689
**Fitness**				
MVPA, min/week	182.8 (151.4)	88.6 (86.8)	224.4 (155.4)	<0.001
Low	73	38	35	
Med	50	9	41	
High	24	1	23	
Missing	14			
**Medication**				
Total number of Medications (mean ± SD)	4.01 (3.44)	8.10 (3.12)	2.15 (1.31)	<0.001
Missing	1			

*Mean (SD) reported. Abbreviations: SD = standard deviation, BMI, body mass index; MEDAS, Mediterranean Diet Adherence Score; MVPA, Moderate to Vigorous Physical Activity*.

Binary logistic regression was used to assess the association between adherence to the MedDiet-score, PA and polypharmacy. Negative binomial regression with a log link function was employed to evaluate the effect of PA, MedDiet-score, age, sex, education level, and BMI on the total number of medications used. Results are reported exponentiated regression coefficients (exp(B)) with 95 % confidence intervals (CI).

Additional logistic regression analyses were conducted to assess associations between PA, MedDiet-score, age, sex, BMI with individual medication groups.

All analyses were performed using IBM SPSS Statistics (Version 29; IBM Corp, Armonk, NY, USA). A two-tailed significance level of *p* < 0.05 was considered statistically significant.

### 2.8 Role of funding source

The MedWalk study, funded by Australia’s National Health and Medical Research Council (NHMRC; GNT1171300), investigates the impact of the Mediterranean diet and exercise on reducing cognitive decline and dementia risk in older adults living independently. However, no additional funding was provided for this study

## 3 Results

A total of 161 participants were included in the study, with a mean age of 74.9 years (SD 5.9). Mean age of participants with polypharmacy was 76.88 years whereas mean age of participants without polypharmacy was 74.09 years. Of the 161 participants, 119 (74 %) were women. The proportion of females was similar between those with polypharmacy (74 %) and those without polypharmacy (74 %), with no significant difference observed (*p* = 0.961). Mean BMI was 31.05 kg/m^2^ and 28.39 kg/m^2^ in participants with and without polypharmacy respectively (*p* = 0.004). The baseline characteristics are shown in Table 1.

### 3.1 Binary logistic regression analysis for polypharmacy

The relationship for polypharmacy (≥5 medications) with PA and MedDiet is shown in Table 2. When compared to participants with low MVPA category, the odds of being a polypharmacy patient were on average 63 % (AOR 0.37 (95 % CI 0.14–0.99); *p* = 0.047) and 92 % (AOR 0.08 (95 % CI 0.01–0.70); *p* = 0.022) lower for participants with moderate and high amounts of MVPA respectively. MedDiet-score was not significantly associated with polypharmacy (AOR 0.97 (95 % CI 0.78–1.19); *p* = 0.738).

**Table 2:** Association of MEDAS and MVPA with polypharmacy*.*

Variable	Odds ratio (OR)	95 % CI for OR		AdjustedOdds ratio (AOR)	95 % CI for AOR	
		Lower limit	Upper limit		Lower limit	Upper limit
MedDiet-score [MEDAS]	0.965	0.810	1.149	0.965*	0.780	1.192
Low physical activity [MVPA]	Ref**	ref	ref	Ref**	ref	ref
Moderate physical activity [MVPA]	**0.238**	**0.101**	**0.561**	0.369**	0.138	0.987
High physical activity [MVPA]	**0.047**	**0.006**	**0.368**	0.081**	0.010	0.696

*Adjusted for age, sex, MVPA, level of education and BMI **Adjusted for age, sex, MEDAS, level of education and BMI. Physical activity (MVPA) categorized as ‘Low’ (<150 min/week of MVPA), ‘moderate’ (150 min/week to <300 min/week of MVPA), or ‘high’ (≥300 min/week of MVPA).

### 3.2 Negative binomial regression analysis for the total number of medications

Univariate analyses show that high PA levels (based on MVPA) was significantly associated with total number of medications used compared to low MVPA (ExpB 0.33 (95 % CI 0.19–0.57); *p* < 0.001). Moderate PA levels and MedDiet were not significantly associated with the total number of medications (resp. ExpB 0.57 (95 % C 0.38–0.85); *p* = 0.006; ExpB 1.01 (95 % CI 0.92–1.11); *p* = 0.882).

Multivariate regression analyses are shown in Table 3. It shows the relationship between total number of medications and age, sex, BMI, level of education, PA, and MedDiet. Regression analysis indicates that high PA levels (based on MVPA) were significantly associated with fewer total number of medications used compared to low MVPA (expB 0.44 (95 % CI 0.22–0.87); *p* = 0.018). Age, sex, BMI, level of education and MedDiet-score were not significantly associated with total number of medications used.

**Table 3:** Multivariate analysis of the association of age, sex, BMI, level of education, categorical score MVPA, and MEDAS with the total number of medication*.*

Variable	Exp(B)	95 % CI for Exp(B)	
		Lower limit	Upper limit
Age (years)	1.024	0.985	1.064
Female	0.828	0.535	1.283
BMI (kg/m2)	1.032	0.990	1.076
Primary school	Ref	Ref	Ref
Secondary school	1.207	0.619	2.353
Diploma	1.225	0.632	2.375
Undergraduate degree	0.938	0.460	1.911
Postgraduate degree	0.879	0.391	1.977
Med-Diet score [MEDAS]	0.997	0.896	1.110
Low physical activity [MVPA]	Ref	Ref	Ref
Moderate physical activity [MVPA]	0.693	0.429	1.117
High physical activity [MVPA]	**0.442**	0.224	0.871
			

Physical activity (MVPA) categorized as ‘Low’ (<150 min/week of MVPA), ‘moderate’ (150 min/week to <300 min/week of MVPA), or ‘high’ (≥300 min/week of MVPA).

### 3.3 Binary logistic regression analysis of specific medication groups

**Fig. 1:**
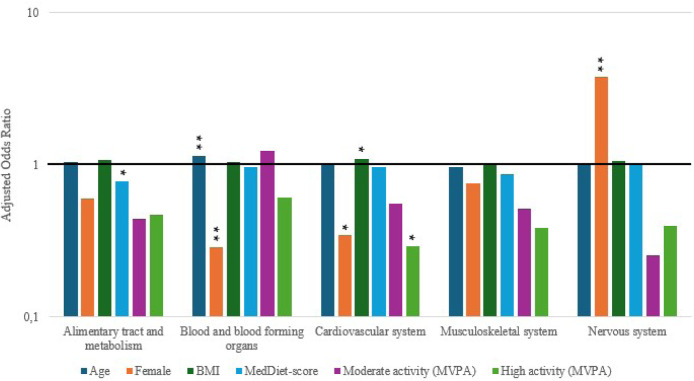
Association of age, sex, BMI, and categorical score MVPA *(*Low’ (<150 min/week of MVPA), ‘moderate’ (150 min/week to <300 min/week of MVPA), or ‘high’ (≥300 min/week of MVPA)) *and MEDAS (1*–*14) with the specific medication groups and y-axis presented as* log scale* *Significance: * p≤0·05, **p≤0·01, ***p≤0·001.

The relationship between the specific type of medication and age, sex, BMI, PA and MedDiet is shown in Fig. 1. Every additional unit (1 point) on the MEDAS was associated with lower use of the medication group prescribed for the alimentary tract and metabolism (AOR 0.78 (95 % CI 0.63–0.96); *p* = 0.016). Those with older age (AOR 1.14 (95 % CI 1.05–1.25); *p* = 0.002) used more medications for blood and blood-forming organs. Female sex was associated with lower use of medications prescribed for blood and blood-forming organs (AOR 0.29 (95 % CI 0.12–0.69); *p* = 0.005). Those with a higher BMI (AOR 1.10 (95 % CI 1.01–1.20); *p* = 0.036) were associated with higher use of the medication group prescribed for the cardiovascular system. Conversely, female sex was associated with lower use of medications prescribed for the cardiovascular system (AOR 0.35 (95 % CI 0.13–0.89); *p* = 0.028). Additionally, the use of medications targeting the cardiovascular system was lower in those who engaged in high amounts of MVPA (≥300 min/week) compared to those with lower amounts of MVPA (<150 min/week) (AOR 0.29 (95 % CI 0.09–0.96); *p* = 0.042). Finally, there was a higher use of medications for nervous system conditions for participants who were female (AOR 3.79 (95 % CI 1.57–9.14); *p* = 0.003).

## 4 Discussion

This study examined the association between adherence to the MedDiet, PA, and medication use in independently living older Australians. The key findings indicate a dose-response relationship between PA and both polypharmacy and total medication use. Specifically, individuals engaging in moderate or high levels of MVPA were less likely to experience polypharmacy compared to those with low MVPA levels. High levels of MVPA was also associated with less medication use in general. And participants with high PA levels used significantly fewer cardiovascular medications compared to those with low PA levels. Contrary to expectations, MedDiet adherence showed no association with total medication use, although higher MedDiet scores were associated with lower use of medications for the alimentary tract and metabolism.

The observed inverse dose-response between MVPA and medication use aligns with prior cross-sectional studies [[21], [22], [23]]. Although literature on PA and polypharmacy in older adults remains limited, a scoping review by Souza et al. identified consistent inverse associations across 14 cross-sectional studies [23]. Similarly, a Brazilian study reported a polypharmacy prevalence of 38.3 % in adults aged ≥60 years, which is comparable to the 40 % found in our sample [21]. Bielemann et al. also observed reduced prevalence of polypharmacy in older adults with higher accelerometer-measured activity levels [21].

Several physiological mechanisms may explain this relationship between PA and medication use. PA is known to prevent chronic disease onset and may act as an adjunct to pharmacotherapy in secondary prevention [24]. Moreover, PA and pharmacological agents often target overlapping molecular and physiological pathways, suggesting that PA might complement or even reduce the need for certain medications. The 2018 Physical Activity Guidelines Advisory Committee (PAGAC) Scientific Report describes a strong linear, inverse relationship between PA and hypertension, with no upper threshold of benefit [6]. For example, each 10 MET-h/week increase in PA is associated with a 6 % reduction in hypertension risk, and typical blood pressure reductions of 2.4–5.2 mmHg systolic and 2.2–4.1 mmHg diastolic have been observed [25]. This may support our findings of lower cardiovascular medication use in participants with higher MVPA.

Higher MedDiet scores were associated with reduced use of medications targeting the alimentary tract and metabolism. This finding is in line with the findings of Esposito et al., who reported improved glycemic control and insulin sensitivity following a MedDiet intervention, potentially reducing the need for medications within the alimentary tract and metabolism group [14].

However, no significant associations were found between MedDiet or PA and medication use for blood and blood-forming organs, the musculoskeletal system, or the nervous system. This was unexpected, given prior evidence linking PA with reduced pain, improved physical function, lower fall risk, enhanced cognitive health, and reduced prevalence of depression and anxiety [6]. Small sample sizes likely limited statistical power. For example, only 45 participants reported use of antithrombotic agents and 20 participants used musculoskeletal drugs, leading to wide confidence intervals. Although AOR for moderate and high MVPA were consistently below 1.0 across several medication classes, statistical significance was not reached. Future studies with larger sample sizes may be able to confirm these associations.

### 4.1 Strengths and limitations

The relationship between the MedDiet, PA, and health outcomes is well-documented, but this study is to our knowledge among the first to examine their association with medication use in Australia. Key strengths include the use of hip-worn accelerometers to provide objective measures of PA, avoiding self-report bias, and the focus on independently living older adults, a group with high potential for positive health interventions.

Several limitations should be considered. Medication use was self-reported, and therefore subject to recall bias. Although participants completed forms at home with medications present, lists may still have been incomplete. Nevertheless, data were sufficiently detailed to enable ATC-code classification, allowing subclass specific analysis. The use of ATC-codes, rather than brand or generic names, further improved comparability across medications. However, regimen adherence and dose information were not assessed, leaving uncertainty as to whether participants took medications as prescribed and in what amounts. This may, in turn, affect outcomes such as engagement in physical activity, for example if medication for musculoskeletal conditions was not used consistently or correctly.

Importantly, the cross-sectional nature of the study means that temporal and causal directions cannot be established. It is equally plausible that healthier individuals take fewer medications and are more active, or that lifestyle behaviors reduce medication need, highlighting the possibility of reverse causality. Residual confounding is also likely, as key variables such as socioeconomic status, comorbidity burden, and medication adherence were not included in the models, despite their plausible influence on both exposures and outcomes.

Due to the exclusion criteria derived from the MEDWALK trial, the generalizability of our findings may be limited. While no participants were excluded for simultaneously reporting participating (on average) in >150 min of moderate-to-vigorous leisure time physical activity per week and a high MedDiet adherence score (defined as a score ≥10 on the 14-item PREDIMED diet questionnaire), exclusion of individuals with neurological conditions may limit conclusions regarding nervous system medications. Similarly, excluding participants unable to walk independently or having major physical ailments that prevent them from regular walking may have constrained findings related to musculoskeletal medications. This reduced variability in key exposures and may underestimate true associations.

Even though objective accelerometry is a strength, they may reflect short-term activity rather than habitual behavior. And because of the cross-sectional nature of the study.duration of lifestyle adherence was not assessed, though these habits often reflect long-term behaviors [26]. The sample was disproportionately female (74 %), consistent with prior studies of older independent-living populations in Australia [27], but limiting sex-based generalizability to older men.

Finally, MedDiet scores were self-reported and showed limited variability (mean = 5.67, SD = 1.93, Q1 = 4, Q3 = 7), with few participants scoring above 9. Which limits the power to detect associations across a wider spectrum of adherence. However this distribution mirrors previous findings in Australian populations, such as Wade et al. (2018), who reported a mean score of 5.4 among adults aged 45–75 years [28], and Chrichton et al. (2013), where 71.1 % of participants scored between 4 and 7 [29]. Broader score distributions in future studies may be necessary to fully evaluate relationships between MedDiet adherence and medication use.

### 4.2 Summary and conclusion

These findings confirm that older Australians living independently who engage in higher PA were observed to use fewer medications and experience less polypharmacy. Higher MedDiet scores were linked to reduced use of alimentary tract and metabolism medications, and more physically active individuals were less likely to use cardiovascular medications.. These findings suggest that maintaining a healthy lifestyle through PA and adherence to a MedDiet may be linked to lower medication use in older adults. Longitudinal and interventional studies are needed to determine whether lifestyle modification through PA or adherence to a MedDiet can reduce polypharmacy and medication burden in later life.

## Funding

This research did not receive any specific grant from funding agencies in the public, commercial, or not-for-profit sectors.

## Disclosures

Over the past 3 years, J.V. has acted as a consultant/advisor for Eisai, KNMP, Med Solutions, Mozand, Red Bull, Sen-Jam Pharmaceutical, and Toast!. The authors declare that the research was conducted in the absence of any commercial or financial relationships that could be construed as a potential conflict of interest.

## CRediT authorship contribution statement

**Lieke Roeke:** Writing – review & editing, Writing – original draft, Data curation, Conceptualization. **Greg Kennedy:** Supervision, Data curation, Conceptualization. **Denny Meyer:** Methodology, Formal analysis. **Michael Kingsley:** Writing – review & editing, Methodology. **Catherine Itsiopoulos:** Writing – review & editing. **Leonie Segal:** Writing – review & editing. **Anne-Marie Minihane:** Writing – review & editing. **Karen J Murphy:** Writing – review & editing. **Tuan Anh Nguyen:** Writing – review & editing, Methodology, Conceptualization. **Jeffery M Reddan:** Writing – review & editing. **Joris C Verster:** Writing – review & editing, Supervision, Conceptualization. **Andrew Pipingas:** Writing – review & editing, Supervision, Conceptualization.

## Declaration of competing interest

The authors declare that they have no known competing financial interests or personal relationships that could have appeared to influence the work reported in this paper.

## Appendix Supplementary materials

Image, application 1

## References

[1] United Nations, Department of Economic and Social Affairs, Population Division (2023).

[2] Australian Institute of Health and Welfare (AIHW) (2022). https://www.aihw.gov.au/reports/australias-health/chronic-conditions-and-multimorbidity.

[3] Department of Health (2015). https://www.health.vic.gov.au/patient-care/medication-and-ageing.

[4] Page A.T., Falster M.O., Litchfield M., Pearson S.A., Etherthon-Beer C. (2019). Polypharmacy among older Australians, 2006–2017: a population-based study. Med J Aust.

[5] Sheikh A., Dhingra-Kumar N., Kelley E., Kieny M.P., Donaldson L.J. (2017). The third global patient safety challenge: tackling medication-related harm. Bull World Health Organ.

[6] US Department of Health and Human Services (2018).

[7] Powell K.E., King A.C., Buchner D.M. (2018). The scientific Foundation for the Physical Activity Guidelines for Americans, 2ndEdition. J Phys Act Health.

[8] Dinu M., Pagliai G., Casini A., Sofi F. (2018). Mediterranean diet and multiple health outcomes: an umbrella review of meta-analyses of observational studies and randomized trials. Eur J Clin Nutr.

[9] Dempsey P.C., Rowlands A.V., Strain T. (2022). Physical activity volume, intensity, and incident cardiovascular disease. Eur Heart J.

[10] Myers J., Kokkinos P., Nyelin E. (2019). Physical activity, cardiorespiratory fitness, and the metabolic syndrome. Nutrients.

[11] Davis C., Bryan J., Hodgson J., Murphy K. (2015). Definiton of the Mediterranean Diet: a literature review. Nutrients.

[12] Pipingas A., Murphy K.J., Davis C.R., Itsiopoulos C., Kingsley M. (2023). A mediterranean diet and walking intervention to reduce cognitive decline and dementia risk in independently living older Australians: the MedWalk randomized controlled trial experimental protocol. J Alzheimers Dis.

[13] WHO (2022). https://atcddd.fhi.no/atc_ddd_index/.

[14] Esposito K., Maiorino M.I., Petrizzo M., Bellastella G., Giugliano D. (2014). The effects of a Mediterranean diet on the need for diabetes drugs and remission of newly diagnosed type 2 diabetes: follow-up of a randomized trial. Diabetes Care.

[15] Sexton C.E., Betts J.F., Demnitz N., Dawes H., Ebmeier K.P., Johansen-Berg H. (2016). A systematic review of MRI studies examining the relationship between physical fitness and activity and the white matter of the ageing bran. NeuroImage.

[16] Martínez-González M.A., Buil-Cosiales P., Corella D. (2019). Cohort Profile: design and methods of the PREDIMED-plus randomized trial. Int J Epidemiol.

[17] Masnoon N., Shakib S., Kalisch-Ellet L., Caughey G.E. (2017). What is polypharmacy? A systematic review of definitions. BMC Geriatr.

[18] Davis C.R., Hodgson J.M., Woodman R., Bryan J., Wilson C., Murphy K.J. (2017). A Mediterranean diet lowers blood pressure and improves endothelial function: results from the MedLey randomized intervention trial. Am J Clin Nutr.

[19] Keadle S.K., Shiroma E.J., Freedson P.S., Lee I.M. (2014). Impact of accelerometer data processing decisions on the sample size, wear time and physical activity level of a large cohort study. BMC Public Health.

[20] Santos-Lozano A., Lugue T., Marín P.J., Ruiz J., Lucia A., Garatachea N. (2012). Intermonitor variability of GT3X accelerometer. Int J Sports Med.

[21] Bielemann R.M., Silveira M.P.T., Lutz B.H. (2020). Objectively measured physical activity and polypharmacy among Brazilian community-dwelling older adults. J Phys Act Health.

[22] Bertoldi A., Hallal P.C., Barros A.J.D. (2006). Physical activity and medicine use: evidence from a population-based study. BMC Public Health.

[23] Souza de I.K.C., Rosa-Souza F.J., Lucena Alves de C.P. (2023). Polypharmacy, physical activity, and sedentary time in older adults: a scoping review. Exp Gerontol.

[24] Giannuzzi P., Mezzani A., Saner H. (2003). Physical activity for primary and secondary prevention. Eur Soc Cardiol.

[25] Liu X., Zhang D., Liu Y. (2017). Dose-response association between physical activity and incident hypertension. Hypertension.

[26] Knoops K.T., Groot de L.C., Kromhout D. (2004). Mediterranean diet, lifestyle factors, and 10- year mortality in elderly European men and women: the HALE project. JAMA.

[27] Australian Bureau of Statistics AG.

[28] Wade A.T., Davis C.R., Dyer K.A., Hodgson J.M., Woodman R.J., Murphy K.J. (2018). A Mediterranean diet supplemented with dairy foods improves markers of cardiovascular risk: results from the MedDairy randomized controlled trial. Am J Clin Nutr.

[29] Crichton G.E., Bryan J., Hodgson J.M., Murphy K.J. (2013). Mediterranean diet adherence and self- reported psychiological functioning in an Australian sample. Appetite.

