# Supplementary material for: The association between medication use and lifestyle factors in independently living older people: impact of Mediterranean diet and physical activity
Source: JAR Life. 2025 Nov 7;14:100041. doi: 10.1016/j.jarlif.2025.100041 (PMC12639465; doi:10.1016/j.jarlif.2025.100041)
Supplement: Supplementary file 1 [file mmc1.docx]

# **Supplemental Material**

**Supplementary table 1. Flowchart study population**


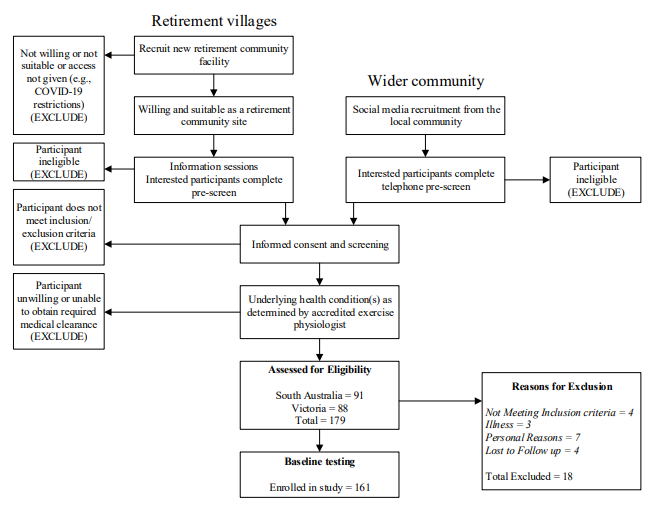


**Supplementary table 2. Medication used by participants with corresponding ATC codes and number of users.**

| **ATC code** | **Medication group** | **Number of users** |
| --- | --- | --- |
| **Alimentary tract and metabolism** | | |
| A01 | Stomatological preparations   - Doxycycline | 4 |
| A02 | Drugs for acid related disorders   - Omeprazole - Pantoprazole - Rabeprazole - Esomeprazole - Alginic acid | 45 |
| A03 | Drugs for functional gastrointestinal disorders   - Mebeverine - Domperidone | 2 |
| A04 | Antiemetics and antinauseants   - Scopolamine | 1 |
| A06 | Drugs for constipation   - Docusate sodium - Senna glycosides, combinations - Ispaghula (psylla seeds) | 4 |
| A07 | Antidiarrheals, intestinal anti-inflammatory/antiinfective agents   - Antibiotics (not recorded which specific one) - Diphenoxylate - Loperamide - Budesonide | 4 |
| A10 | Drugs used in diabetes   - Insulin (not recorded which specific one) - Insulin aspart - Insulin degludec and insulin aspart - Insulin glargine - Metformin - Gliclazide - Metformin and sitagliptin - Linagliptin and empagliflozin - Metformin and empagliflozin - Sitagliptin - Linagliptin - Dulaglutide - Semaglutide - Dapagliflozin - Empagliflozin | 37 |
| **Blood and blood forming organs** | | |
| B01 | Antithrombotic agents   - Warfarin - Clopidogrel - Acetylsalicylic acid - Dabigatran etexilate - Rivaroxaban - Apixaban | 51 |
| **Cardiovascular system** | | |
| C01 | Cardiac therapy   - Digoxin - Flecainide - Glyceryl trinitrate - Isosorbide mononitrate | 19 |
| C02 | Antihypertensives   - Clonidine - Moxonidine - Prazosin | 7 |
| C03 | Diuretics   - Hydrochlorothiazide - Chlortalidone - Indapamide - Furosemide - Spironolactone - Hydrochlorothiazide and potassium-sparing agents | 19 |
| C07 | Beta blocking agents   - Propranolol - Sotalol - Metoprolol - Atenolol - Nebivolol | 20 |
| C08 | Calcium channel blockers   - Amlodipine - Felodipine - Nifedipine - Lercanidipine - Verapamil - Diltiazem - Perhexiline | 27 |
| C09 | Agents acting on the renin-angiotensin system   - Lisinopril - Perindopril - Ramipril - Trandolapril - Valsartan - Irbesartan - Candesartan - Telmisartan - Olmesartan medoxomil - Irbesartan and diuretics - Candesartan and diuretics - Telmisartan and diuretics - Olmesartan medoxomil and amlodipine - Telmisartan and amlodipine - Valsartan, amlodipine and hydrochlorothiazide - Valsartan and sacubitril | 73 |
| C10 | Lipid modifying agents   - Simvastatin - Pravastatin - Atorvastatin - Rosuvastatin - Fenofibrate - Ezetimibe - Simvastatin and ezetimibe - Atorvastatin and ezetimibe - Rosuvastatin and ezetimibe - Atorvastatin and amlodipine | 78 |
| **Musculo-skeletal system** | | |
| M01 | Anti-inflammatory and antirheumatic products   - Diclofenac - Meloxicam - Ibuprofen - Naproxen - Celecoxib | 6 |
| M04 | Antigout preparations   - Allopurinol | 2 |
| M05 | Drugs for treatment of bone diseases   - Risedronic acid - Denosumab | 12 |
| **Nervous system** | | |
| N02 | Analgesics   - Oxycodone - Pethidine - Buprenorphine - Codeine and paracetamol - Tramadol - Tapentadol - Acetylsalicylic acid - Paracetamol - Sumatriptan - Rizatriptan - Pizotifen - Pregabaline | 42 |
| N03 | Antiepileptics   - Primidone - Valproic acid - Lamotrigine - Levetiracetam | 5 |
| N04 | Anti-parkinson drugs   - Pramipexole | 1 |
| N05 | Psycholeptics   - Prochlorperazine - Lurasidone - Quetiapine - Diazepam - Temazepam - Zopiclone - Melatonin | 18 |
| N06 | Psychoanaleptics   - Amitriptyline - Citalopram - Sertraline - Escitalopram - Moclobemide - Mirtazepine - Venlafaxine - Duloxetine - Agomelatine - Desvenlafaxine - Atomoxetine | 37 |
| N07 | Other nervous system drugs   - Betahistine | 4 |

**Pain medications such as analgesics and NSAIDs may fall under different ATC categories depending on their primary indication and formulation. Classification in this table is based on both WHO ATC code [13] and particpants’ self-reported reasons for medication use.*

**Supplementary table 3. MedWalk Mediterranean Diet Assessment (MW-MEDAS).^16,17^**

| **Foods and frequency of consumption** | **Criteria for 1 point^1^** |
| --- | --- |
| 1. Do you use extra virgin olive oil as your main culinary fat? (please circle Y or N) | Y |
| 1. How many tablespoons of extra virgin olive oil do you have per day? | ≥ 3 Tbsp^2^ |
| 1. How many serves of vegetables do you have per day? (1 serving = ½ cup cooked vegetables or 1 cup salad) | ≥ 5 |
| 1. Do you use sofrito sauce, a sauce of olive oil, onions, tomato and garlic, at least twice per week? | 1 |
| 1. How many serves of fruit, including fresh, dried and tinned fruit, do you have per day? (1 serve = 1 piece of fruit or 1 handful dried) | ≥ 2 |
| 1. How many servings of Greek yoghurt and cheese do you have per day? (1 serve = 2/3 cup yoghurt, 2 slices hard cheese or 3 slices soft cheese) | 2-4 |
| 1. How many servings of breads, cereals or other grain foods do you have per day? (Include all bread, cereals, crispbreads, rice, pasta, couscous, quinoa, porridge, oats and muesli). (1 serve = 1 slice bread, 1/3 cup cereal, ½ cup cooked rice or pasta) | 3-6 |
| 1. Do you include at least half of your grains and cereals as whole meal or whole grain? | Y |
| 1. How many servings of fish do you have per week (1 serve = 1 small fillet, 4 prawns) | ≥ 3 |
| 1. How many serves of legumes do you have per week? (1 serve = ½ cup cooked) | ≥ 3 |
| 1. How many servings of nuts do you have per week? Include nuts you use in cooking or salads. (1 serve = 1 handful) | ≥ 5 |
| 1. How many servings of red and processed meat do you have per week? (1 serve = 100 g red meat, 50 g processed meat (e.g. bacon, ham, salami, sausages) | ≤2 |
| 1. How many glasses of red wine do you have per week? (1 glass = 100 ml, or 1 small glass) | ≤14 serves^3^ |
| 1. How many times per week do you eat discretionary foods, such as chocolate, biscuits, lollies, cakes, deep fried foods, soft drink, non-red wine alcoholic beverages, sweet pastries or other desserts? | ≤3 |

^1^ Criterion to score 1 point. Otherwise, 0 recorded.

^2^ 1 tablespoon = 20mL

^3^ 1 serve = 100 mL
